# Strategies of zooplanktivory shape the dynamics and diversity of littoral plankton communities: a mesocosm approach

**DOI:** 10.1002/ece3.1488

**Published:** 2015-04-16

**Authors:** Laura K Helenius, Anna Aymà Padrós, Elina Leskinen, Hannu Lehtonen, Leena Nurminen

**Affiliations:** 1 Department of Environmental Sciences, University of HelsinkiP.O. Box 65, Helsinki, 00014, Finland; 2 Tvärminne Zoological Station, University of HelsinkiJ. A. Palménin tie 260, Hanko, 10900, Finland; 3 Institute of Marine Sciences (ICM-CSIC)Passeig Marítim de la Barceloneta 37-49, Barcelona, 08003, Spain; 4 GRC Marine Geosciences, Department of Marine Stratigraphy, Paleontology and Geosciences, University of BarcelonaCarrer Martí i Franquès s/n, Barcelona, 08028, Spain

**Keywords:** Baltic Sea, feeding strategy, *Gasterosteus aculeatus*, mesocosm, *Rutilus rutilus*, zooplankton

## Abstract

Planktivorous fish can exert strong top-down control on zooplankton communities. By incorporating different feeding strategies, from selective particulate feeding to cruising filter feeding, fish species target distinct prey. In this study, we investigated the effects of two species with different feeding strategies, the three-spined stickleback (*Gasterosteus aculeatus* (L.)) and roach (*Rutilus rutilus* (L.)), on a low-diversity brackish water zooplankton community using a 16-day mesocosm experiment. The experiment was conducted on a small-bodied spring zooplankton community in high-nutrient conditions, as well as a large-bodied summer community in low-nutrient conditions. Effects were highly dependent on the initial zooplankton community structure and hence seasonal variation. In a small-bodied community with high predation pressure and no dispersal or migration, the selective particulate-feeding stickleback depleted the zooplankton community and decreased its diversity more radically than the cruising filter-feeding roach. Cladocerans rather than copepods were efficiently removed by predation, and their removal caused altered patterns in rotifer abundance. In a large-bodied summer community with initial high taxonomic and functional diversity, predation pressure was lower and resource availability was high for omnivorous crustaceans preying on other zooplankton. In this community, predation maintained diversity, regardless of predator species. During both experimental periods, predation influenced the competitive relationship between the dominant calanoid copepods, and altered species composition and size structure of the zooplankton community. Changes also occurred to an extent at the level of nontarget prey, such as microzooplankton and rotifers, emphasizing the importance of subtle predation effects. We discuss our results in the context of the adaptive foraging mechanism and relate them to the natural littoral community.

## Introduction

Predation has the potential to significantly shape communities, yet its actual impact in natural conditions is challenging to estimate experimentally because of the complex species interactions and interferences in real food webs (e.g., Micheli 1999; Blumenshine and Hambright 2003). Direct effects of fish predation on zooplankton communities have been studied extensively, often with an emphasis on the depletion of focal crustacean prey (Chang et al. 2004; Hansson et al. 2007). Both marine and freshwater food webs are characterized by top-down control of mesozooplankton through predation by fish (Brooks and Dodson 1965; Hall et al. 1976; Micheli 1999). By entirely eliminating mesozooplankton in small freshwater systems, planktivorous fish can also indirectly elevate densities of small-bodied rotifers, microzooplankton, and phytoplankton via a trophic cascade (Hurlbert and Mulla 1981; Brett and Goldman 1996). High predation pressure by zooplanktivores can cause zooplankton communities to be completely top-down controlled, so that resource availability no longer counts as a factor in shaping the community (Nicolle et al. 2011).

Fish utilize different strategies of zooplanktivory according to their physical capacities and focal prey type. Visual detection of prey is a key issue for selective particulate feeders, which tend to attack an individual planktonic prey species, and prey switching occurs when the relative abundance of profitable prey changes due to predation (Lazzaro 1987). Specific detection is not necessary for cruising filter-feeders, as they forage by engulfing a volume of water containing several prey items (Lazzaro 1987; Lammens and Hoogenboezem 1991). Laboratory observations suggest that juvenile cyprinids, such as roach (*Rutilus rutilus* (L.)), are able to forage by both particulate and filter-feeding modes depending on prey size and density (Lammens 1985; Lammens et al. 1987). The three-spined stickleback (*Gasterosteus aculeatus* (L.)) on the other hand is an obligate vision-oriented and selective particulate feeder, which uses a pause-travel foraging strategy that is distinct from the constant rapid swimming which the rounded fusiform body of the roach is adapted for (Keast and Webb 1966; Wootton 1976, 1984; Lazzaro 1987; Tudorache et al. 2008). These two common zooplanktivorous key species often coexist in coastal areas and interspecific competition through diet overlap may occur. Disparate feeding strategies have been shown to target different prey species (e.g., Estlander et al. 2010), which raises questions about their potentially divergent effects on the diversity of prey populations.

Predators are known to alter their resource choice in the face of changing resource abundance (e.g., Oaten and Murdoch 1975). In a complex food web, the average number of prey available per predator can be high (e.g., Woodward et al. 2005). The adaptive food web hypothesis (Kondoh 2003) suggests adaptive foraging as a mechanism that promotes stability in these complex food webs. How the prey number changes with altered food web complexity depends on the ability of the predator to forage adaptively: A nonadaptive forager, such as a cruising filter-feeding roach, allocates its foraging effort to all potential prey species, while an adaptive forager, such as the selective particulate-feeding stickleback, may consume only a fraction of the potential prey species, as those of low quality or quantity are discarded from the diet. Adaptive foraging then creates a positive stability–complexity relationship by deterring extinction by consumption, where alternative resources cause a predation shift to another prey species when target prey abundance becomes low (Kondoh 2005). We are interested in the consequences of this possible mechanism on zooplankton community structure and diversity.

Diversity can be discussed either as a taxonomic or a functional measure. To measure the functional diversity of a community, organisms are grouped based on common attributes instead of taxonomy. The ecological roles of organisms, as opposed to merely numbers of taxonomic species present, are important when considering the relationship between biodiversity and ecosystem function (Hooper and Vitousek 1997; Symstad et al. 2000). Species that are seemingly functionally redundant can contribute to ecosystem resilience when environmental conditions change. Functional traits of species describe their response to or effect on the environment, and if these are known, accurate predictions can be made on community shifts in the face of environmental change (Barnett et al. 2007). The Baltic Sea is unique in its low biodiversity compared to other marine areas (e.g., Elmgren 1984), making it particularly appropriate for studying structuring factors such as predation. In such structurally simple ecosystems, the significance of single species may increase, as they can be responsible for performing multiple functions (Bonsdorff and Pearson 1999).

The aim of this study was to examine the successional dynamics of a low-diversity brackish water zooplankton community modified by fish predators with different feeding strategies. Using low levels of predation pressure, we created a closed system mesocosm governed by resources, thus ensuring that we detected the effects of foraging without depleting the zooplankton community too rapidly or completely. We studied how taxonomic and functional zooplankton diversity changed during a 16-day period and how seasonal variation in community composition influenced the outcome. We did this by conducting the experiment separately in a small-bodied spring community and a large-bodied summer community.

We expected zooplanktivorous fish to reduce mesozooplankton in general. We formulated our hypotheses based on the concept of adaptive foraging and expected (1) the stickleback to act as a size-selective (adaptive) forager and shift the community size structure in favor of smaller species and (2) the roach to act as an efficient, nonselective (nonadaptive) forager, depleting the community more evenly with only a minor effect on community size structure. In terms of species composition, we hypothesized that (3) predation would enhance succession of rotifers,inhibit succession of cladocerans, and affect existing species interactions by inhibiting the influence of competition and resource limitation. We expected (4) diversity to be maintained by the adaptive foraging of the stickleback and to be decreased by the nonadaptive foraging of the roach.

## Methods

### Mesocosm construction and sampling

The two study periods took place in June (spring community) and August (summer community) of 2012, with experimental periods lasting 16 days. Nine UV-resistant 2225-L polyethylene enclosures (1.5 m × 1.5 m × 1.5 m) were placed at a depth of 0.9–1.1 m in a shallow bay in the vicinity of the Tvärminne Zoological Station (TZS) (Hanko, SW coast of Finland, northern Baltic Sea) and filled with 2000 L of surrounding seawater. The plankton community in each enclosure consisted of a mixture of the natural surrounding seawater community and additional zooplankton acquired from the nearby Storfjärden area, to maximize both density and diversity of the experimental community. The additional zooplankton was collected with vertical and horizontal hauls from depths of 1.5 to 15 meters using plankton nets of 100 to 200 *μ*m mesh size. The mixture created was calculated to be equivalent to ten times the current natural density of zooplankton. Equal aliquots of the plankton mixture were added to each enclosure and allowed to settle for 5 h before fish were released into the enclosures.

All fish were caught with a beach seine from the vicinity of the TZS and acclimated for at least 12 h before being released into the enclosures. Three fish were released into each of six enclosures, so that three enclosures contained sticklebacks and three contained roach. We arrived at this fish density from sampling littoral areas with a beach seine to determine approximate fish amounts in natural conditions. An additional three fishless enclosures served as controls, giving a total of three treatments. The enclosures were sampled before releasing the fish (day 1) and on days 4, 10, and 16 of the 16-day experiment. Fish body lengths were measured after the experiment and ranged from 6.4 to 8.0 cm with no significant difference between the two fish species (one-way ANOVA, *F*_1,16_ - 2.149, *P *-* *0.162).

Sampling was conducted using a 2.85-L Limnos water sampler. The water in each enclosure was gently mixed to minimize effects of patchy distribution, and a total of 8.55 L of water was removed. The water was sieved through a 25-*μ*m plankton net and the samples were immediately fixed with 5% acid Lugol's solution. An additional 800 mL was removed from each enclosure for water chemistry measurements (total nitrogen [TN], total phosphorus [TP], chlorophyll *a* [Chl *a*], turbidity and salinity), and water temperature was recorded.

To determine algal biomass (expressed as Chl *a*), 200 mL of water was filtered (GFF filter, 25 mm) no later than 12 h after sampling and the filters frozen until further analysis. Chlorophyll *a* was extracted from the filter using 5 mL of ethanol and the solution read with a spectrophotometer. Turbidity (in nephelometric turbidity units, NTU) was determined using a standard turbidity meter (Hach 2100P; Hach Co., Loveland, CO), and salinity (VWR EC300 Portable conductivity, salinity and temperature instrument) and pH (Jenway 3510 Bibby Scientific ltd., Staffordshire, UK) were measured. Nutrient concentrations were determined according to methods by Koroleff (1979).

Because of high densities, the zooplankton samples were halved into subsamples using a Folsom plankton splitter. Subsamples were filtered through netting material, washed into a volume of 10 mL into a cylindrical settling chamber, and allowed to settle for 30 min into a single-unit counting chamber. Using a phase-contrast microscope, all individuals were identified and counted in one subsample, and crustaceans as well as individuals of dominant rotifer groups (only *Synchaeta* spp. in the spring and additionally *Keratella* spp. in the summer) were measured. Only crustaceans were identified, counted, and measured in the other subsample. Individuals were identified to the lowest possible taxonomical level and life stages of copepods were documented as calanoid/cyclopoid nauplii, copepodites, or adults, where only adults were identified to the species or genus level.

### Data analysis

Two-factor ANOVA with repeated measures (RMA) was used to compare differences in water temperature and algal biomass between enclosures, and the *t*-test was used to compare initial nutrient values between spring and summer periods. Species/group abundances were calculated as individuals L^−1^. Mean weighted mesozooplankton size (crustaceans and dominant rotifers) for both spring and summer communities was calculated to estimate community size structure as follows:

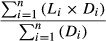


where *L*_*i*_ is the mean length of species *i* in a sample and *D*_*i*_ is the density of species *i* in that sample. One-way ANOVA was used to compare differences in mean weighted size between initial spring and summer communities, and RMA was used to compare patterns of change in size between treatments throughout the experiments. RMA was used to compare patterns of total zooplankton abundance and zooplankton group abundances separately (microzooplankton, rotifers, cladocerans, and adult copepods) in each treatment. Pairwise comparisons were conducted with the Holm–Sidak correction. When a significant time*treatment interaction was found through RMA, sampling days were separately examined with a one-way ANOVA and post hoc tests (Tukey's HSD).

Multivariate analyses were used to explore zooplankton data. Abundances were square-root-transformed to minimize the effect of dominant species and to include the effect of rarer ones. Transformed data were used to generate a Bray–Curtis similarity matrix and calculate a partly nested permutational MANOVA (PERMANOVA) (Sams and Keough 2012) to test for differences in overall community structure between treatments. PERMANOVA was followed by pairwise comparisons when significant differences were detected between treatments (*P*-value based on either PERMANOVA or Monte Carlo (*MC*) methods in cases of low sample size and few unique permutations). Nonmetric multi-dimensional scaling (nMDS) graphics and principal coordinate ordinations (PCO) were used to visualize differences in overall community structure through time. The similarity percentages procedure (SIMPER) was used to determine which genera/species contributed most to the Bray–Curtis dissimilarities between samples.

The Shannon–Weaver diversity index (*H′*) was used as a measure of taxonomic diversity. Functional diversity values were calculated for mesozooplankton from trait data. Traits were chosen to reflect resource use and included feeding type, trophic group based on prey type, and prey size range. Qualitative measures were entered as rank categories. A functional dendrogram was generated using hierarchical clustering analysis, resulting in five functional groups of crustaceans with similar effects on trophic transfer. Functional diversity (FD sensu Petchey and Gaston 2002) values were calculated as the total branch length needed to join all genera in an assemblage. RMA was used to compare patterns in FD and *H′* values.

For each ANOVA and RMA model, assumptions were examined for normality, tested for homogeneity of variance, and data were transformed if needed. For each RMA, the Greenhouse–Geisser adjustment of *P*-values was used as a conservative estimate of probability to compensate for any violation of sphericity. SPSS v 21 (IBM 2012) and PRIMER v 6 (PRIMER-E Ltd., Plymouth, UK) were used to analyze the data.

## Results

### Physical and chemical analysis

**Figure 1:**
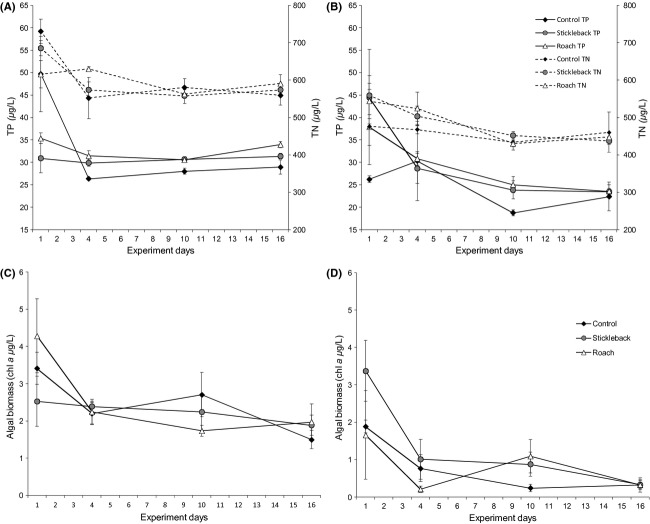
Nutrient concentrations (total phosphorus [―] and total nitrogen [---]) in each enclosure in spring (A) and summer (B) experiments, and algal biomass (measured as chl *a*) in each enclosure in spring (C) and summer (D) experiments. Error bars are ± SE. Correction added on 28 April 2015, after first publication: The legend and axis titles ‘TN’ and ‘TP’ were previously indicated wrongly as “NT” and “PT” and have now been corrected in this version.

Salinity (range 5.1–5.7 psu), turbidity (range 2–6 NTU), and pH (range 7.4–8.5) values remained constant throughout both experimental periods. Water temperature in the spring period was lower than in the summer period, with means (±SE) of 13.4 ± 0.4°C and 17.6 ± 0.6°C, respectively. Temperature rose during the experimental periods along with air temperature but did not significantly differ between treatment enclosures at either time period (RMA, time*treatment effect, *P *>* *0.05). Total nutrients were on average higher in the spring than in the summer (*t*-test, *t*(35) - 2.25 (TP) and 9.21 (TN), *P *<* *0.05 for both) (Fig.1A and B). Initial nutrient values varied but stabilized during the experimental periods and remained slightly higher in predator enclosures. This may have been due to the recycling of nutrients by fish. Algal biomass varied throughout the experimental periods but stabilized at a low value on day 16 (Fig.1C and D).

### Community size structure and abundance

**Figure 2:**
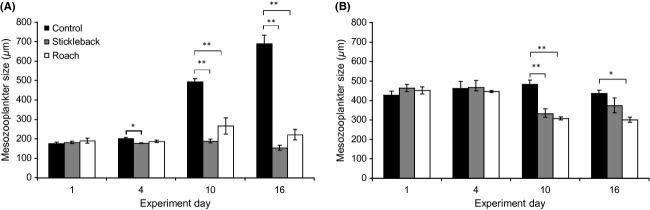
Mean weighted size of the average mesozooplankter (rotifers and crustaceans) in spring (A) and summer (B) experiments. Asterisks indicate significant differences (*P <* *0.05, **P <* *0.01) between treatments. Error bars are ± SE.

During the spring period, the mean size of the average mesozooplankter in the initial community was significantly influenced by time as well as treatment, as expected. However, as was indicated by the significant time*treatment interaction, the changes in size structure throughout the experiment were very heterogeneous between treatments (Table1; Fig.2A). Mean weighted size in the control was significantly higher than in the stickleback enclosure or both predator enclosures on sampling days 4, 10, and 16 (*F*_2,6_ - 6.819, *P *<* *0.05, *F*_2,6_ - 37.07, *P *<* *0.001, *F*_2,6_ - 97.03, *P *<* *0.001, respectively). In the absence of predation, community size structure changed toward larger species, and the mean weighted size had increased over threefold by day 16, while in the predator enclosures, size decreased slightly (stickleback) or remained roughly the same (roach) (Fig.2A).

**Table 1:** Summary of the results of a two-factor ANOVA with repeated measures (RMA) for the analysis of differences in weighted size, total abundance, microzooplankton, rotifer, cladoceran, and copepod abundance across time and predator treatment in the spring and summer periods

		Spring			Summer		
	Source of variation	df	*F*	*P*	df	*F*	*P*
Weighted size	Time	3	67.761	**<0.001**	3	11.313	**<0.001**
	Treatment	2	81.558	**<0.001**	2	2.036	0.211
	Time^*^treatment	6	57.005	**<0.001**	6	5.702	**0.002**
Total abundance	Time	3	5.654	**0.007**	3	27.771	**<0.001**
	Treatment	2	27.282	**0.001**	2	5.141	**0.05**
	Time^*^treatment	6	0.905	0.513	6	3.376	**0.021**
Microzoopl. abundance	Time	3	11.871	**0.01**	3	3.257	0.121
	Treatment	2	12.107	**0.008**	2	0.316	0.740
	Time^*^treatment	6	4.592	0.051	6	0.372	0.706
Rotifer abundance	Time	3	20.282	**<0.001**	3	40.424	**<0.001**
	Treatment	2	5.90	**0.038**	2	7.393	**0.024**
	Time^*^treatment	6	1.098	0.401	6	7.495	**0.015**
Cladoceran abundance	Time	1.7	1.864	0.206	3	2.214	0.122
	Treatment	2	13.377	**0.006**	2	4.576	0.062
	Time^*^treatment	3.4	6.078	**0.01**	6	2.676	**0.049**
Copepod abundance	Time	3	23.236	**<0.001**	1.4	8.556	**0.014**
	Treatment	2	29.157	**0.001**	2	2.383	0.173
	Time^*^treatment	6	18.383	**<0.001**	2.8	4.409	**0.041**

Values significant at the 0.05 level are in bold

**Figure 3:**
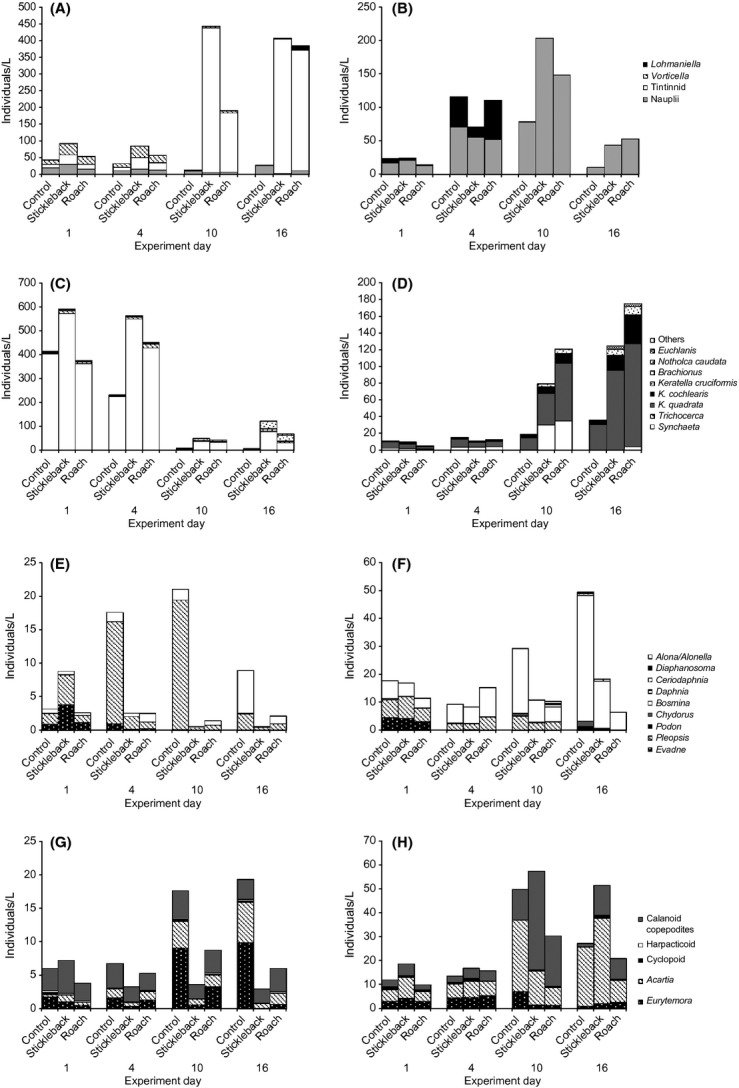
Mean total abundances of zooplankton in each treatment (control, stickleback, roach) on days 1, 4, 10, and 16 of spring (A, C, E, and G) and summer (B, D, F, and H) experiments, showing proportions of species/genera in the community: microzooplankton and nauplii (A, B), rotifers (C, D) cladocerans (E, F), and copepods (G, H). Note different axis scales.

In the spring, initial total abundance of individuals was high. Both time and treatment determined total abundance, which decreased overall on day 10 (Table1). Pairwise comparisons revealed that the stickleback enclosures had a significantly higher total abundance of zooplankton than the other enclosures, while the control had the lowest abundance (*P *<* *0.05). High total abundance was reflected in a high abundance of small-bodied zooplankton groups, such as microzooplankton and rotifers, while low total abundance was associated with a high abundance of larger crustaceans (Fig.3A, C, E, and G). Microzooplankton and rotifer abundances were also determined by time and treatment (Table1). The stickleback enclosures had a significantly higher abundance of these groups than the control (pairwise comparisons *P *<* *0.01 and *P *<* *0.005, respectively), but the rotifer population collapse on day 10 was common to all treatments. Meanwhile, patterns in cladoceran and copepod abundance varied between treatments, as shown by the significant time*treatment interactions (Table1). Crustacean abundance in predator enclosures was significantly lower than in the control toward the end of the experiment (day 10 *F*_2,8_ - 23.033, *P *<* *0.005; and day 16 *F*_2,8_ - 9.124, *P *<* *0.05 for cladocerans; day 10 *F*_2,8_ - 19.423, *P *<* *0.005; and day 16 *F*_2,8_ - 136.416, *P *<* *0.001 for copepods) (Fig.3E and G).

In the summer period mean weighted size of the initial community was significantly higher than in the spring period (*F*_1,16_ - 486.19, *P *<* *0.001) (Fig.2B). The changes in size were more moderate than during the spring period, but as in the spring, the trajectory of the changes varied between treatments, as indicated by the significant time*treatment interaction (Table1). In predator enclosures, weighted size decreased on day 10. Size in the control remained the same and differed significantly from both predator enclosures on day 10 (*F*_2,6_ - 22.78, *P *<* *0.005) but only from roach enclosures on day 16 (*F*_2,6_ - 6.74, *P *<* *0.05) (Fig.2B).

Initial group abundances in the summer reflected the large-bodied community, with lower abundances of microzooplankton and rotifers and higher abundances of crustaceans compared to the spring period (Fig.3B, D, F, and H). Total zooplankton abundance increased in all enclosures with time, as opposed to the decreasing trend in the spring, but the response pattern differed with treatment, as indicated by the significant time*treatment interaction (Table1). On day 16, total abundance was significantly higher in predator enclosures than in the control (*F*_2,8_ - 13.125, *P *<* *0.01). This was due to rotifer abundance, which was significantly higher in both predator enclosures toward the end of the experiment, although to a lesser extent in the stickleback enclosures on day 16 (*F*_2,6_ - 16.755, *P *<* *0.005 on day 10 and *F*_2,6_ - 5.855, *P *<* *0.05 on day 16). Similarly to the spring experiment, the significant time*treatment interaction revealed that the succession of cladocerans and adult copepods differed between treatments (Table1). Adult copepods were slightly more abundant in stickleback enclosures throughout the experiment, except on day 10, when abundance was significantly higher in the control (*F*_2,8_ - 13.862, *P *<* *0.01) (Fig.3H). For cladocerans, no significant differences between treatments could be found due to high variation, despite the significant interaction term.

### Zooplankton community: spatial and temporal differences in composition

**Figure 4:**
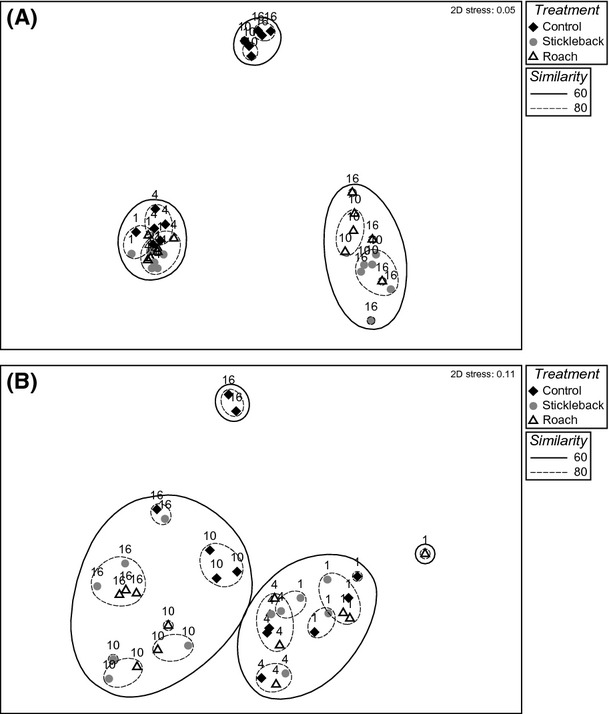
NMDS ordination of zooplankton communities (square-root-transformed data) in (A) spring (2D stress - 0.05) and (B) summer (2D stress - 0.11) experiments. Numbers represent sampling days 1, 4, 10, and 16. Superimposed clusters are based on Bray–Curtis similarities at levels of 60% (solid line) and 80% (dashed line).

**Figure 5:**
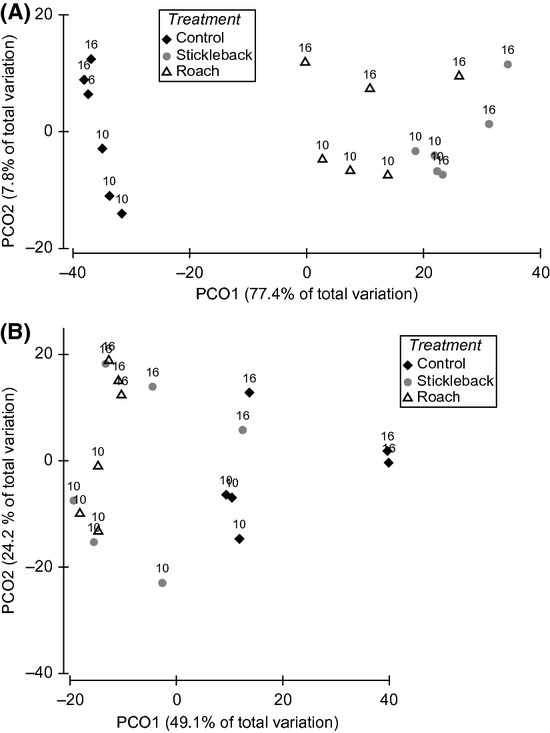
Principal coordinates ordination (PCO) of distances among samples on the basis of square-root-transformed Bray–Curtis measures of taxa abundance at the last two sampling dates (days 10 and 16) in (A) spring and (B) summer experiments.

The initial spring and summer communities differed in species composition, with the spring community consisting of higher abundances of microzooplankton and rotifers, and the summer community consisting of threefold higher abundances of crustaceans (Fig.3). Throughout the duration of the spring experiment, there were clear differences in zooplankton succession between treatments, as indicated by the significant time*treatment interaction of the partly nested PERMANOVA (*F *-* *6.60, *P *-* *0.001) and segregation of stickleback enclosures from the control as early as the fourth experimental day (pairwise comparisons *t *-* *2.52, *P*(*MC*) < 0.05) (Figs.3A, C, E, G and 4A). According to the PCO, 77.4% of the variation in samples of day 10 and day 16 was explained by the first axis, which classified samples according to treatment, while only 7.8% was explained by the axis, which classified samples according to time (Fig.5A). By day 10, predator enclosures clearly differed from the control, with 80% similarity between control samples (pairwise comparisons stickleback *t *-* *5.36, *P*(*MC*) - 0.001 and roach *t *-* *4.29, *P*(*MC*) < 0.005) (Fig.4A). The SIMPER analysis revealed that the difference between the predator enclosures and the control was caused mainly by abundances of *Tintinnopsis lobiancoi*, *Synchaeta,* and *Pleopsis polyphemoides*, which together contributed 55–60% of the dissimilarity (Fig.3A, C, and E; Fig. S1). *Eurytemora* also contributed 5% of the dissimilarity between stickleback and control enclosures (Fig.3G). A difference between predator enclosures was also detectable, as roach and stickleback enclosures were segregated into their own groups, with 80% similarity within groups (pairwise comparisons *t *-* *1.93, *P*(*MC*) < 0.05). The SIMPER analysis showed that the main difference between roach and stickleback enclosures was caused by divergent abundances of *T. lobiancoi*, *Vorticella*, and *Eurytemora*, which together contributed to 48% of the dissimilarity (Fig.3A and G; Fig. S1).

On day 16 of the spring experiment, the predator enclosures were more variable within treatments and more homogenous between treatments, with 60% similarity between all samples regardless of predator type (Fig.4A). Both stickleback and roach enclosures were characterized by *T*. *lobiancoi*, *Synchaeta*, and *Keratella cruciformis*, which together contributed up to 80% of the similarity within stickleback enclosures but only 55% of the similarity within roach enclosures. Roach enclosures were more diverse and additionally typified by calanoid nauplii, *K*. *quadrata*, *Notholca*, calanoid copepodites, and *Acartia*. The dissimilarity between the predator treatments was mostly caused by higher abundances of *T. lobiancoi* and *Synchaeta* and lower abundances of calanoid nauplii in stickleback enclosures compared to roach enclosures (S1). Both predator enclosures remained significantly segregated from the control (pairwise comparisons stickleback *t *-* *6.03, *P *-* *0.001, roach *t *-* *3.63, *P*(*MC*) < 0.005), in which typical groups were calanoid nauplii, *Eurytemora*, and *Acartia*, together contributing up to 45% of the total similarity of 82%.

Comparable to the spring, zooplankton succession did not coincide between treatments in the summer period, as indicated by the significant time*treatment interaction (*F *-* *2.30, *P *<* *0.001). All the communities were similar in the first two sampling days, but from day 10 onwards, predator enclosures differed from the control (pairwise comparisons stickleback *t *-* *2.32, *P*(*MC*) < 0.05 and roach *t *-* *2.74, *P*(*MC*) < 0.01) (Fig.4B). Average similarity between predator enclosures and the control (65.5%) decreased below similarity within control enclosures (81%), but there was no such segregation between predator enclosures. The PCO suggested that almost half of the variation in communities of day 10 and day 16 was explained by treatment (Fig.5B). According to the SIMPER analysis, calanoid nauplii were typical in all enclosures, with *Keratella quadrata* typical in predator enclosures and *Acartia* and *Bosmina* typical in the control (Fig.3B, D, F, and H). Higher abundances of *Synchaeta* and calanoid copepodites and a lower abundance of *Bosmina* in stickleback enclosures compared to the control inflicted 50% of the dissimilarity between them (Fig.3D, F, and H; Fig. S2). The corresponding percentage dissimilarity between roach enclosures and the control was due to considerably lower abundances of *Synchaeta*, *K*. *quadrata,* and calanoid nauplii and a higher abundance of *Acartia* in the former compared to the latter (Fig.3B, D, and H; Fig. S2). On day 16, roach enclosures were very homogenous (84% similarity) and least similar to the control (53% similarity, pairwise comparisons, *t *-* *3.32, *P*(*MC*) < 0.05), which also significantly differed from stickleback enclosures (pairwise comparisons, *t *-* *2.25, *P*(*MC*) < 0.05). The cladoceran *Podon leuckartii* was unique to the control. The dissimilarity between enclosures was mainly due to higher abundances of *K. quadrata*, *K. cochlearis,* and calanoid nauplii and a lower abundance of *Bosmina* in predator enclosures compared to the control (Fig.3B, D, and F; Fig. S2). There was a low average dissimilarity (24%) between the predator treatments, which was mainly caused by the high abundance of *Acartia* in stickleback enclosures (Fig.3H).

### Diversity

**Figure 6:**
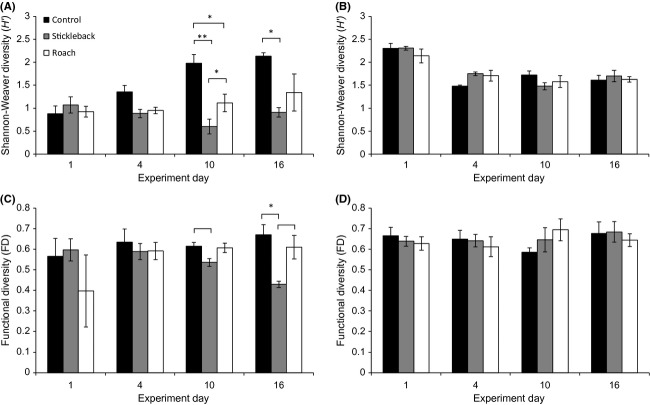
Means of Shannon-Diversity index (*H*') of all taxa in spring (A) and summer (B) experiments, and functional diversity index (FD) of crustaceans in spring (C) and summer (D) experiments. Indices are plotted over the four sampling days, with brackets indicating marginally significant (*P *<* *0.08) and asterisks indicating significant differences (*P <* *0.05, **P <* *0.01) between treatments. Error bars are ± SE.

The *H′* values were calculated including all encountered taxa. Initial spring values were similar in all enclosures (*F*_2,6_ - 0.428, *P *>* *0.05). However, a significant time*treatment interaction showed that the pattern of response differed between treatments. On the last sampling days, *H′* was significantly lower in stickleback enclosures than in the other treatments (day 10 *F*_2,6_ - 231.893, *P *<* *0.001 and day 16* F*_2,6_ - 6.455, *P *<* *0.05) (Table2; Fig.6A). The time*treatment interaction was also significant for functional diversity (FD) (Table2; Fig.6C). On day 16, FD was significantly lower in the stickleback than control enclosures, in direct opposition of the initial sampling (*F*_2,6_ - 7.011, *P *<* *0.05). The significant decline on day 16 was due to an almost complete lack of cladocerans in stickleback enclosures (Fig.3E). Cyclopoids, *Eurytemora,* and harpacticoids, which composed two functional groups, were also essentially missing from stickleback enclosures, but were present in roach enclosures and in the control (Fig.3G). In the summer period, unlike in the spring, predation had no effect on either diversity measure. *H′* values decreased significantly in all enclosures, with no treatment effect (Table2; Fig.6B).

**Table 2:** Summary of the results of a two-factor ANOVA with repeated measures (RMA) for the analysis of differences in two measures of diversity (the Shannon–Weaver diversity index, *H′*, and functional diversity, FD) across time and predator treatment in the spring and summer periods

		Spring			Summer		
	Source of variation	df	*F*	*P*	df	*F*	*P*
Taxonomic diversity (*H*′)	Time	3	6.363	**0.004**	3	28.988	**<0.001**
	Treatment	2	48.561	**<0.001**	2	0.303	0.750
	Time^*^treatment	6	5.188	**0.003**	6	1.510	0.231
Functional diversity (FD)	Time	3	1.580	0.229	3	0.458	0.715
	Treatment	2	1.350	0.328	2	0.029	0.972
	Time^*^treatment	6	3.197	**0.026**	6	0.966	0.475

Values significant at the 0.05 level are in bold

## Discussion

In the spring period, stickleback predation appeared to decrease community size structure, while roach predation merely prevented a large increase in size, as hypothesized. In a community initially dominated by rotifers, predation efficiently controlled structural development by removing large crustaceans. Size-selective predation has recurrently been shown to shift the size structure of zooplankton communities in favor of smaller species (Brooks and Dodson 1965; Hall et al. 1976; Carpenter and Kitchell 1996; Brett and Goldman 1997; Hansson et al. 2007). An incoming dispersal and simultaneous eradication of large-bodied crustaceans by predation generally enables small species to invade the community (Shurin 2001). As expected, this effect was visible in the stickleback enclosures, where large crustaceans were the individually sought target prey, whereas the overall low crustacean density was likely to encourage feeding on smaller prey by cruising roach. Filter-feeding cyprinids are efficient zooplanktivores, but they tend to capture prey with poor motility or inferior swimming abilities (Persson 1987). Hence, prey availability and escape ability were more important than size in shaping the community in roach enclosures, and average zooplankter size did not decrease.

In the summer period, the initial community was crustacean-dominated, and overall predation effects were more obvious than in the spring. Instead of merely suppressing the development of the community toward larger body size, predation actively decreased mean prey size in both predator enclosures due to peaks in densities of small species (*Synchaeta, Keratella,* and nauplii). The late summer rotifer abundance peak that occurred in predator enclosures is also seen in the natural Baltic Sea system, suggesting that predation effects in our mesocosms were comparable to those in real ecosystems (Scheinin and Mattila 2010). Like in the spring, the smaller size classes of zooplankton benefited from the presence of fish, as was hypothesized. Zooplankton size, rather than biomass, generally responds predictably to planktivory (Pace 1984; Soranno et al. [Bibr b501]). However, changes in size structure alone did not show conclusive differences between the two predators in either spring or summer.

On the timescale of 16 days, most of the variation in the zooplankton communities was attributable to changes in the abundances of a few key taxa. In the predator enclosures, a microzooplankton group (*T. lobiancoi* in the spring and calanoid nauplii in the summer) underwent a rapid population surge, while a rotifer species underwent either a crash (*Synchaeta* in the spring) or an abrupt rise (*K*. *quadrata* in the summer). The known natural zooplankton succession of corresponding Baltic littoral areas is similar: Calanoid nauplii are generally dominant in the abundance peaks in mesotrophic environments, and *Keratella* characterizes these sites later on in the season (Scheinin and Mattila 2010). Densities of the cladocerans *Pleopsis polyphemoides* in the spring and *Bosmina* in the summer were maintained low compared to the control. Of the above groups only the cladocerans are known as distinct target prey for zooplanktivores. Evidently, the influence of predators on lower trophic levels can be more subtle than simple resource exploitation, because the mere presence of predatory fish can induce reduced foraging behavior in prey organisms (Englund 2005). Such behavioral cascades imply that predator–prey behavioral interactions can alter ecosystem processes at the base of aquatic food webs (Townsend 2003; Byrnes et al. 2006). In our experiments, such possible cascades were detected in the abundances of tintinnids and nauplii, which increased to a greater extent in stickleback than in roach enclosures. More detailed experimentation could reveal whether these are examples of feeding linkages severed upon predator manipulation (Krivan and Schmitz 2003).

Predator enclosures generally had higher rotifer and lower cladoceran densities than the control during both experimental periods. The population dynamics of these groups are characterized by extreme oscillations in abundance: Typically cladocerans and rotifers are capable of explosive population growth and equivalently rapid crashes (Likens 2010). Herbivorous cladocerans are known to suppress rotifers through mechanical interference or exploitative competition, especially species of *Synchaeta*, *Keratella,* and *Trichocerca* (Gilbert 1989). As expected, rotifers in the predator enclosures underwent enhanced succession in the absence of large cladocerans and other crustaceans, including the copepods *Thermocyclops*, *Mesocyclops,* and *Acartia*, which have all been shown to readily ingest rotifers such as *Synchaeta* (Egloff 1988; Nagata and Hanazato 2006). As fish predation removed larger competitors and potential predators, rotifers rapidly populated the newly produced vacant niches due to their fast reproductive rates (Nogrady et al. 1993). The appearance of *Keratella cruciformis* in the spring and the shift from *Synchaeta* to *Keratella* in the summer were indications of rapid rotifer succession, as *Keratella* tends to succeed the more aggressively feeding raptorial *Synchaeta* in Baltic Sea coastal systems (e.g., Scheinin and Mattila 2010). *Synchaeta* is mainly found during periods of high phytoplankton production (Heinbokel et al. 1988) and a decrease in algal biomass in the enclosures may have benefited other rotifer species. Meanwhile, abundances of *Synchaeta* and *Keratella* remained low in control enclosures, where they were presumably kept in check by large crustaceans.

The low densities of cladocerans in the predator enclosures inflicted a large part of the dissimilarity found between the predator enclosures and the control, because large crustacean zooplankton (e.g., *Acartia* and *P*. *polyphemoides*) are strongly predator controlled (Horsted et al. 1988). Cladoceran vulnerability or preference over copepod prey has been demonstrated for both roach and sticklebacks (e.g., Winfield et al. 1983; Helenius et al. 2013); hence, no significant differences between the predators were observed concerning cladocerans. The substantial population increases of *P. polyphemoides* in the spring and *Bosmina* in the summer occurred only in the control and were clearly inhibited by predation, regardless of feeding strategy. In the summer period, the replacement of *Pleopsis* with the larger *Podon leuckartii* was observed only in the control. This was expected, as predation is considered to keep populations at densities where exploitative competition is not significant enough to cause such species replacement (Gliwicz and Pijanowska 1989). In an extensive study of zooplankton in the littoral area surrounding our mesocosms, Scheinin and Mattila (2010) also found *P. leuckartii* to be unique to a specific mesotrophic site, suggesting that it may require environmental conditions that are rarely met, for example, low or nonexistent predation.

The main compositional difference in target prey densities between the two predators was observed as a change in the competitive interaction between the calanoid copepods, *Eurytemora* and *Acartia*, presumably caused by selective feeding by sticklebacks. *Eurytemora* has higher food ingestion rates and probable higher growth efficiency; therefore, it has the potential to be more numerous than *Acartia* when food resources are adequate (Adrian et al. 1999), and it clearly dominated the spring control. However, stickleback foraging appeared to influence this interaction, as demonstrated by the near extinction of *Eurytemora* in the stickleback enclosures. *Eurytemora* is expected to be the preferred prey for particulate feeders because of its larger size, and egg-carrying females are often targeted by visual predators (Rajasilta and Vuorinen 1983; Viitasalo et al. 2001). *Acartia* is less conspicuous because of its smaller size and the females’ egg depositing behavior (Viitasalo et al. 2001). The extinction of *Eurytemora* was concurrent with recent studies on interactions between predation and resource availability. With high predation pressure, resource competition becomes irrelevant and predation alone controls the prey population (Nicolle et al. 2011). In the roach enclosures, *Acartia* had also become the dominant copepod by day 16, but without *Eurytemora* extinction. Predation by cruising feeders does not target *Eurytemora*, but *Acartia* is more sensitive to hydrodynamic disturbance, and this “alertness” and the resulting lower predator encounter rate probably render it less vulnerable to filter-feeding roach (Viitasalo et al. 2001).

The competitive interaction between the two dominant calanoids was affected by predation in the summer period as well, but in a slightly different manner than in the spring. *Acartia* was clearly more abundant than *Eurytemora* in the control enclosures, in direct reversal to the spring experiment. The same pattern has been observed in the field (Adrian et al. 1999). The lower phytoplankton availability of the summer period conceivably favored *Acartia*, which has a wider food niche resulting from its unique capacity for raptorial as well as suspension feeding (Gyllenberg 1980; Tiselius 1990). Stickleback predation should theoretically enhance the difference in relative calanoid abundances, because *Eurytemora* is expected to be more susceptible to selective predation, and this was seen in the high *Acartia* abundance in the last sampling day. *Acartia* also contributed most to the existing community dissimilarity between the predator enclosures, implying that predation efficiency on *Acartia* was higher in the roach enclosures. Here, we must consider the possibility of a switch in the roach feeding strategy in the summer period. Theoretically, the large body size of the average zooplankter encourages particulate feeding in a species that is capable of both strategies (Lammens 1985; Lammens et al. 1987). This was supported by the altogether minimal differences between the predator enclosures in the summer period, as well as the effective decline in size structure in roach enclosures, which is a conceivable indication of size-selective feeding and, therefore, incongruent with our hypothesis.

In direct opposition to the spring community, the high rotifer abundance and low copepod abundance were more exaggerated in the roach enclosures in the summer, again suggesting that predation pressure was higher than in the stickleback enclosures. Relative amounts of species/genera were similar on all sampling days, with the exception of the high abundance of *Acartia* in stickleback enclosures on day 16. Increased resources in the form of rotifers and nauplii presumably buffered the effects of stickleback predation on *Acartia*, which had doubled in abundance by day 16. When crustaceans were readily available, predation by the stickleback seemed to target *Bosmina* and *Eurytemora*, while the cruising roach evenly and efficiently controlled all available groups, thereby not allowing any particular group to dominate the community.

Dominance by a few species tends to be exacerbated in a stressed ecosystem (e.g., Warwick and Clarke 1993). In the spring, the stickleback enclosures became dominated by two rotifer species and the tintinnid *T. lobiancoi*, which indicated high predation pressure in the community. Both taxonomic and functional diversity decreased in the stickleback enclosures in the spring period, while it increased (*H′*) or remained the same (FD) in the roach enclosures, in direct opposition to our hypothesis. Even with the stickleback acting as an adaptive forager, initial diversity in the spring community was not sufficiently high to maintain, and without incoming dispersal that would allow small species to populate newly formed niches, predation merely removed species and depleted diversity. Foraging by roach actually increased diversity on this time scale, as it did not cause near extinctions of large crustaceans (*Bosmina*, *Eurytemora*) or intense domination by small plankters.

Conversely in the summer period, when the initial community was more diverse and made up of larger plankters, we did not observe effects on diversity measurements by either predator. Diversity itself regulates predation, because the species not included in a predator's diet can weaken predator–prey interactions by masking prey, diluting prey concentrations, or confusing predators (Kratina et al. 2007). Both functional and taxonomic diversities were already high in the summer period, possibly weakening the zooplanktivore–prey link. Taxonomic diversity decreased in all enclosures during the experiment, when some taxonomic groups became dominant. Even so, functional redundancy overrid the effects of species loss, which rendered FD values stable. Hence, predation was not a factor in an already diverse community.

Typically, there is no obvious decline in ecosystem functioning when species disappear, but once a whole guild of functionally identical species is lost, there may be a dramatic collapse (Woodward 2009). This scenario was depicted in the FD values of the spring stickleback enclosures when an entire functional group was lost at the disappearance of the cyclopoids *Mesocyclops* and *Thermocyclops*, which formed a functional group as omni-carnivorous raptorial feeders with large prey. Due to their sporadic abundance, their significance for taxonomic diversity was low. Yet cyclopoids have been shown to efficiently control rotifer populations (Nagata and Hanazato 2006) and they may have largely shaped the disparate *Synchaeta* abundances in the spring predator enclosures. Cladocerans and herbivorous copepods disappeared from stickleback enclosures, which may also have strong implications on grazing processes. Crustaceans graze on different size classes, and cladocerans essentially control biomasses of small phytoplankton (Sommer and Sommer 2006). Unlike in the stickleback enclosures, the discrepancy in taxonomic diversity between roach enclosures and the control in the spring period was not reflected in functional diversity. Although crustacean abundances were markedly lower in roach enclosures, functional group composition remained similar to control enclosures, further corroborating that cruising predation by roach is nonselective by nature.

We conclude that the effect of feeding by these fish predators largely depends on initial zooplankton community structure and hence seasonal variation in a temperate system. The adaptive foraging hypothesis can be useful in interpreting changes in prey communities, but making generalizations on the effects of different feeding strategies would require further observations using a larger array of fish species. This study emphasizes the importance of examining subtle changes in zooplankton communities, such as those occurring on nonprey species, as a response to predation. More focus should be addressed to indirect compositional and relative changes in species abundance, because very little is known about how these subtle changes might alter ecosystem functioning.

## Supporting Information

**Table S1.** Dissimilarities between treatments on days 10 and 16 of the experiment (spring period), displaying species/genera which cumulatively contribute to over 50% of the dissimilarity determined from the SIMPER analysis.

**Table S2.** Dissimilarities between treatments on days 10 and 16 of the experiment (summer period), displaying species/genera which cumulatively contribute to over 50% of the dissimilarity determined from the SIMPER analysis.
