# Supplementary material for: Strategies of zooplanktivory shape the dynamics and diversity of littoral plankton communities: a mesocosm approach
Source: Ecol Evol. 2015 Apr 16;5(10):2021–35. doi: 10.1002/ece3.1488 (PMC4449756; doi:10.1002/ece3.1488)
Supplement: Supplementary file 1 [file ece30005-2021-sd1.docx]

| Day | | Treatment pair | | Dissimilarity percentage | | Species/group | Percentage contribution |
| --- | --- | --- | --- | --- | --- | --- | --- |
| 10 | Control – Stickleback | | 58.56% | | *Tintinnopsis lobiancoi* (micro)  *Synchaeta* spp. (rot) | | 41.57% 10.14% |
| 10 | Control – Roach | | 47.58% | | *Tintinnopsis lobiancoi* (micro)  *Synchaeta* spp. (rot)  *Pleopsis polyphemoides* (clad) | | 33.04% 12.36% 10.02% |
| 10 | Stickleback – Roach | | 22.30% | | *Tintinnopsis lobiancoi* (micro)  *Vorticella* spp. (micro) *Eurytemora* spp. (cop)  cyclopoid nauplius (micro) | | 37.75% 5.25%  4.84% 4.70% |
| 16 | Control – Stickleback | | 71.57% | | *Tintinnopsis lobiancoi* (micro)  *Synchaeta* spp. (rot)  *Keratella cruficormis* (rot) | | 33.66%  14.13%  7.83% |
| 16 | Control – Roach | | 54.54% | | *Tintinnopsis lobiancoi* (micro)  *Synchaeta* spp. (rot)  *Keratella cruciformis* (rot) | | 34.98%  10.63%  9.24% |
| 16 | Stickleback – Roach | | 29.05% | | *Tintinnopsis lobiancoi* (micro)  *Synchaeta* spp. (rot)  calanoid nauplius (micro) *Lohmaniella* spp. (micro)  *Notholca* spp. (rot) | | 24.07%  10.98%  6.65%  6.23%  5.75% |
