# Supplementary material for: Strategies of zooplanktivory shape the dynamics and diversity of littoral plankton communities: a mesocosm approach
Source: Ecol Evol. 2015 Apr 16;5(10):2021–35. doi: 10.1002/ece3.1488 (PMC4449756; doi:10.1002/ece3.1488)
Supplement: Supplementary file 2 [file ece30005-2021-sd2.docx]

| Day | Treatment pair | Dissimilarity percentage | Species/group | | Percentage contribution |
| --- | --- | --- | --- | --- | --- |
| 10 | Control – Stickleback | 34.52% | | Calanoid nauplius (micro)  *Synchaeta* spp. (rot)  Calanoid copepodite (cop*)*  *Bosmina* spp. (clad) | 16.55%  15.92%  9.37%  8.79% |
| 10 | Control – Roach | 34.48% | | *Synchaeta* spp. (rot)  *Keratella quadrata* (rot)  Calanoid nauplius (micro)  *Acartia* spp. (cop) | 15.30%  15.10%  10.82%  9.02% |
| 10 | Stickleback – Roach | 22.21% | | Calanoid nauplius (micro)  *Keratella quadrata* (rot)  Calanoid copepodite (cop)  *Synchaeta* spp. (rot)  *Bosmina* spp. (clad)  *Pleopsis polyphemoides* (clad) | 11.94%  10.70%  10.22%  9.73%  6.61%  5.46% |
| 16 | Control – Stickleback | 41.27% | | *Keratella quadrata* (rot)  Calanoid nauplius (micro)  *Bosmina* spp. (clad)  *Keratella cochlearis* (rot)  Calanoid copepodite (cop) | 16.05%  11.70%  11.01%  8.48%  7.46% |
| 16 | Control – Roach | 47.12% | | *Keratella quadrata* (rot)  Calanoid nauplius (micro)  *Keratella cochlearis* (rot)  *Bosmina* spp. (clad) | 17.03%  11.58%  11.49%  11.43% |
| 16 | Stickleback – Roach | 23.85% | | *Acartia* spp. (cop)  *Keratella quadrata* (rot)  *Keratella cochlearis* (rot)  *Bosmina* spp. (clad)  *Synchaeta* spp. (rot)  *Euchlanis dilatata* (rot) | 14.26%  12.01%  8.98%  7.81%  6.46%  5.48% |

Table S2. Dissimilarities between treatments on days 10 and 16 of the experiment (summer period), displaying species/genera which cumulatively contribute to over 50% of the dissimilarity determined from the SIMPER analysis. Species/genera are grouped as microzooplankton (micro), rotifers (rot), cladocerans (clad) and copepods (cop).
